# Complement component 5 promotes lethal thrombosis

**DOI:** 10.1038/srep42714

**Published:** 2017-02-16

**Authors:** Tomohiro Mizuno, Kengo Yoshioka, Masashi Mizuno, Mie Shimizu, Fumihiko Nagano, Tomoyuki Okuda, Naotake Tsuboi, Shoichi Maruyama, Tadashi Nagamatsu, Masaki Imai

**Affiliations:** 1 Department of Analytical Pharmacology, Meijo University, Nagoya, Japan; 2 Department of Renal Replacement Therapy, Nagoya University, Nagoya, Japan; 3 Department of Enviromental Sciences, Meijo University, Nagoya, Japan; 4 Yokohama Brain and spine Center, Yokohama, Japan; 5 Department of Drug Delivery Research, Meijo University, Nagoya, Japan; 6 Department of Nephrology, Nagoya University, Nagoya, Japan; 7 Department of Immunology, Nagoya City University, Nagoya, Japan

## Abstract

Extracellular histones promote platelet aggregation and thrombosis; this is followed by induction of coagulation disorder, which results in exhaustion of coagulation factors. Complement component 5 (C5) is known to be associated with platelet aggregation and coagulation system activation. To date, the pathological mechanism underlying liver injury has remained unclear. Here, we investigated whether C5 promotes liver injury associated with histone-induced lethal thrombosis. C5-sufficient and C5-deficient mice received single tail vein injections of purified, unfractionated histones obtained from calf thymus (45–75 μg/g). Subsequently, the mice were monitored for survival for up to 72 h. Based on the survival data, the 45 μg/g dose was used for analysis of blood cell count, liver function, blood coagulation ability, and promotion of platelet aggregation and platelet/leukocyte aggregate (PLA) production by extracellular histones. C5-deficient mice were protected from lethal thrombosis and had milder thrombocytopenia, consumptive coagulopathy, and liver injury with embolism and lower PLA production than C5-sufficient mice. These results indicate that C5 is associated with coagulation disorders, PLA production, and embolism-induced liver injury. In conclusion, C5 promotes liver injury associated with histone-induced lethal thrombosis.

Disseminated intravascular coagulation (DIC) is seen in patients with severe sepsis and acute promyelocytic leukaemia^1^. In these patients, systemic inflammation activates the coagulation system and induces coagulation factor consumption^2^. These responses induce lethal thrombosis, thereby leading to a poor prognosis^3^. In DIC, cellular injury can release neutrophil extracellular traps^4^,^5^, neutrophil elastase^4^,^6^, myeloperoxidase^4^, and histones^5^,^7^. Plasma histone levels are elevated in patients with septic^8^ and non-septic DIC^9^. Extracellular histones promote platelet aggregation^10^, neutrophil migration^11^,^12^, and thrombosis^10^, followed by induction of hypercoagulation^10^ and hyperfibrinolysis, resulting in exhaustion of coagulation factors^8^,^11^,^13^. Furthermore, extracellular histones induce liver damage via release of inflammatory cytokines^14^.

The complement system plays important roles in innate immunity and protection of the host from pathogens. However, unregulated complement activation causes severe inflammation^15^,^16^. Recent studies have shown molecular intercommunication between complement and coagulation fibrinogen lysis systems^17^,^18^,^19^,^20^. Thrombin^17^ and factors IX, X, and XI promote complement component 5 (C5) cleavage^21^. Clark *et al*. reported that plasmin also contributes to C5 cleavage via non-traditional complement activation^18^. In turn, the coagulation cascade is promoted by activation of the complement system^19^,^20^,^22^,^23^. Recently, it has been shown that complement component 3a (C3a) and complement component 4d (C4d) are deposited on the platelet surface in trauma patients and promote platelet aggregation^24^. Thrombotic microangiopathy, including haemolytic uremic syndrome (HUS), is a systemic syndrome that is characterized by platelet aggregation and promotes liver injury^25^. Anti-C5 therapy is used for HUS^26^,^27^,^28^. These previous reports indicate that C5 is associated with platelet aggregation and coagulation system activation. Although the mechanisms underlying histone-induced thrombosis and platelet aggregation have been elucidated by previous reports^10^,^11^,^13^, the pathological mechanism underlying liver injury has remained unclear. Therefore, in the present study, we investigated whether C5 promotes liver injury associated with histone-induced lethal thrombosis.

## Results

### C5-deficient mice were protected from lethal thrombosis

**Figure 1:**
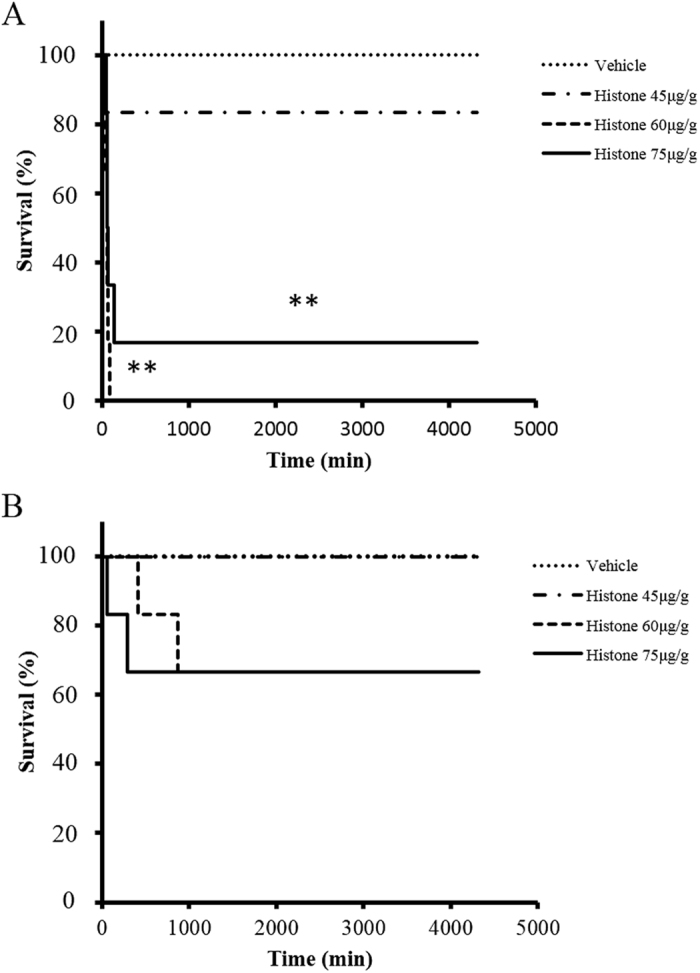
Mortality after administration of histone. Mice were injected with histone (45–75 μg/g, *n* = 6 per group) or saline. Survival curves of C5-sufficient or deficient are shown in figure (**A** or **B**), respectively. **P < 0.01 (log-rank test).

The data pertaining to mortality after histone administration are shown in Fig. 1. Histone dose-dependently induced lethal thrombosis. In the case of C5-sufficient mice, survival rates of the high-dose group (≧60 μg/g) were significantly lower than those of the vehicle group (Fig. 1A). C5-deficient mice were protected from histone-induced lethal thrombosis (Fig. 1B), but C5-sufficient mice were not.

### Thrombocytopenia and consumptive coagulopathy were milder in C5-deficient mice than in C5-sufficient mice

**Figure 2:**
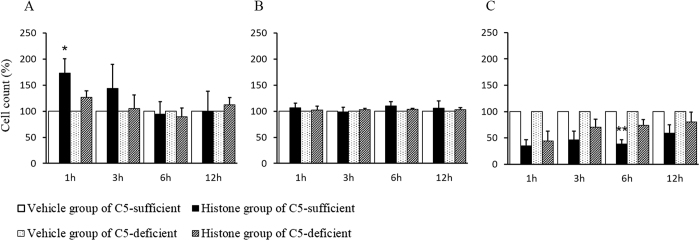
Extracellular histones induced thrombocytopenia. Mice were injected with histone or saline (45 μg/g, *n* = 6 per group). The blood samples were collected at 1–12 h after injection. The numbers of white blood cells (WBCs), red blood cells (RBCs), and platelets are shown in figure (**A**–**C**), respectively. Data are presented as percentage of the vehicle group. The figures show the experimental means ± SD. *P < 0.05, **P < 0.01 vs Histone group of C5-deficient (Scheffe’s test).

The blood cell counts are shown in Fig. 2. The white blood cell (WBC) counts in C5-sufficient mice increased 1 and 3 h after histone injection (Fig. 2A). The red blood cell (RBC) counts did not significantly differ between the C5-sufficient and C5-deficient mice (Fig. 2B). The platelet counts decreased in both types of mice; however, the degree of thrombocytopenia in C5-deficient mice was lesser than that in C5-sufficient mice (Fig. 2C).

**Figure 3:**
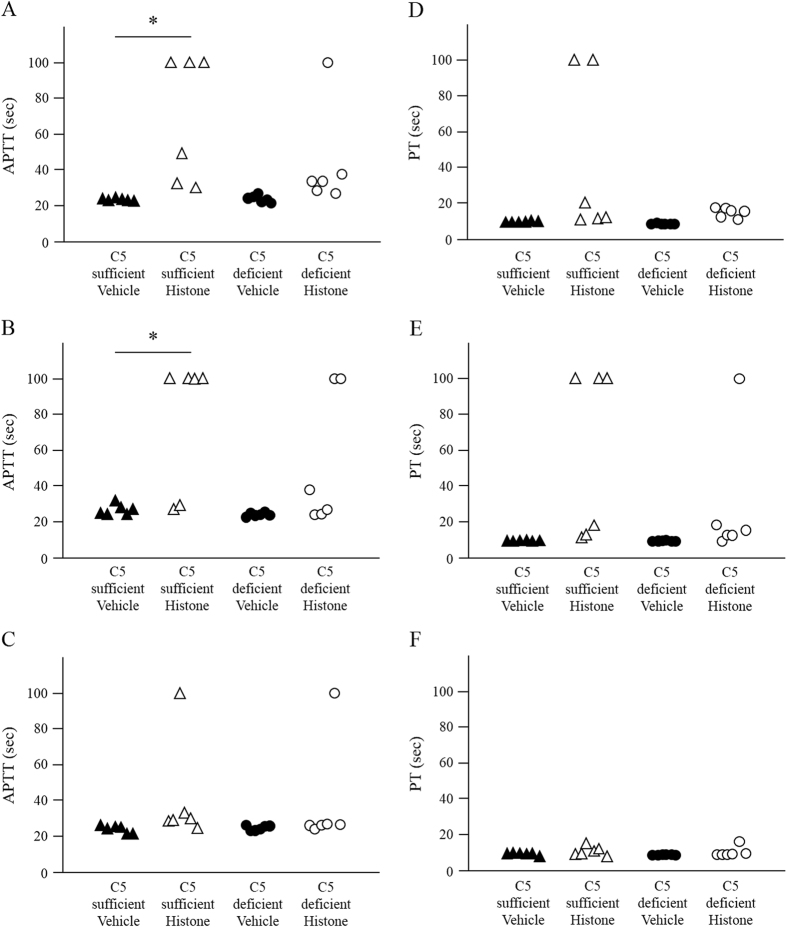
Extracellular histones prolonged plasma activated partial thromboplastin time (APTT) and prothrombin time (PT) in C5-sufficient mice. Mice were injected with histone or saline (45 μg/g, *n* = 6 per group). The blood samples were collected at 1–6 h after injection. APTT at 1, 3 and 6 h are shown in figure (**A**–**C**), respectively. PT at 1, 3 and 6 h are also shown in (**D**–**F**), respectively. *P < 0.05 vs Vehicle group of C5 sufficient (Scheffe’s test).

Data for activated partial thromboplastin time (APTT) and prothrombin time (PT) are provided in Fig. 3. In C5-sufficient mice, the APTTs 1 and 3 h after histone injection were significantly prolonged as compared to those in the vehicle-treated mice (1 h: *p* = 0.024, 3 h: *p* = 0.036) (Fig. 3A and B). However, there were no significant differences in C5-deficient mice at any time point. Furthermore, C5-deficient mice recovered from consumptive coagulopathy 6 h after histone injection.

### Liver injury and embolism were more severe in C5-sufficient mice than in C5-deficient mice

**Figure 4:**
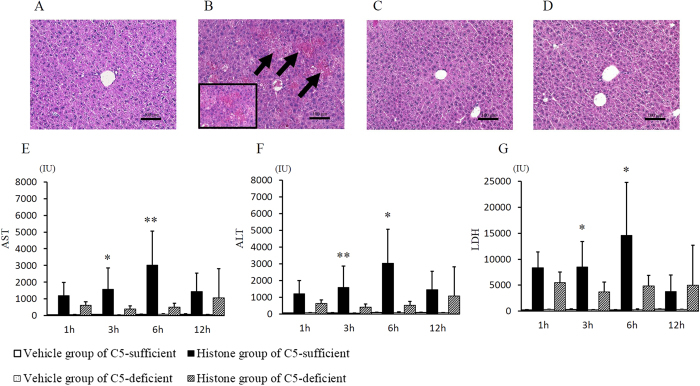
Extracellular histones induced severe liver dysfunction. Mice were injected with histone or saline (45 μg/g, *n* = 6 per group). Histological findings are show in fig. (**A** to **D**) at 6 h after histone injection. (**A**) Vehicle group of C5-sufficient, (**B**) Histone group of C5-sufficient, (**C**) Vehicle group of C5-deficient, (**D**) Histone group of C5-deficient. H&E stain. Scale bar, 100 μm. Black allows indicates embolism. The blood samples were collected at 1–6 h after histone injection. Liver function were defined as AST, ALT and LDH levels, and these are shown in (**E**–**G**), respectively. The figures show the experimental means ± SD. *P < 0.05, **P < 0.01 vs Histone group of C5-deficient (Scheffe’s test).

**Figure 5:**
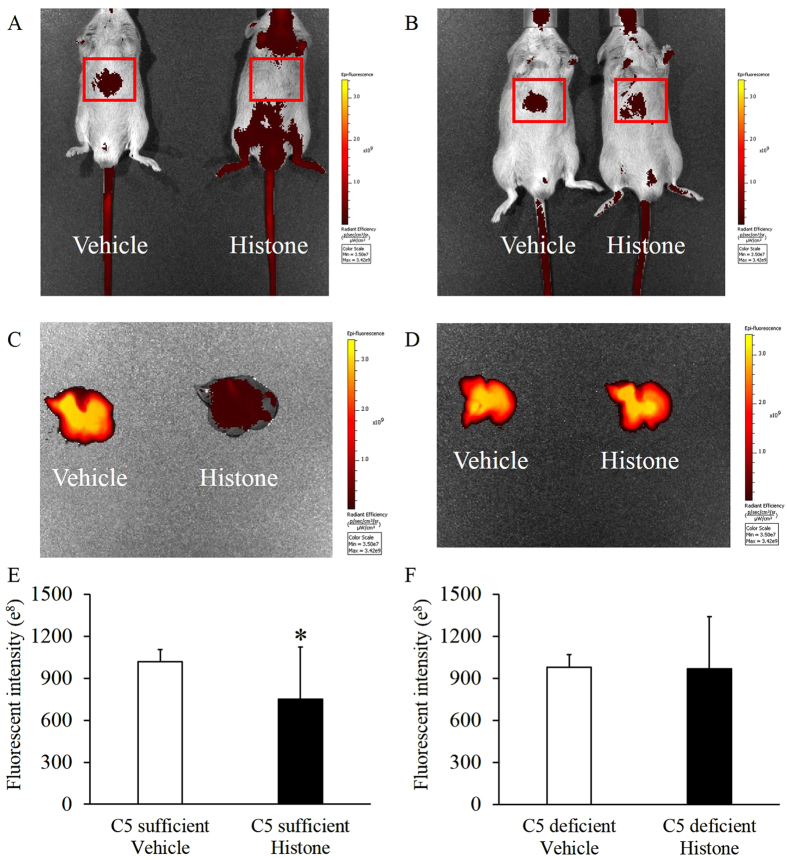
Extracellular histones decreased hepatic blood flow in C5-sufficeint mice. Mice were injected with histone or saline (45 μg/g, *n* = 6 per group). Mice were administrated to indocyanine green (ICG) at 1 h after injection with histone. The images were acquired at 10 min after intravascular injection of ICG. Liver tissues were collected from same mice. (**A**) Image of C5-sufficient mice, (**B**) Image of C5-deficient mice, (**C**) Image of liver tissue in C5-sufficient mice, (**D**) Image of liver tissue in C5-deficient mice, (**E**) Fluorescent intensity of liver tissue in C5-sufficient mice, (**F**) Fluorescent intensity of liver tissue in C5-deficient mice. The figures show the experimental means ± SD. *P < 0.05 vs Vehicle group of C5-deficient (Student’s-t test).

Pathological analysis 6 h after histone injection showed the presence of embolism in C5-sufficient mice (Fig. 4A to D). The AST, ALT, and LDH levels are shown in Fig. 4. The levels in C5-sufficient mice were significantly higher than those in C5-deficient mice 3–6 h after the histone injection (Fig. 4E to G). Moreover, indocyanine green (ICG) did not accumulate in the liver in C5-sufficient mice that received the histone injection, whereas ICG accumulated in C5-deficient mice (Fig. 5A to D); the ICG intensity significantly decreased in the liver of C5-sufficient mice (Fig. 5E). These results showed that the hepatic blood flow decreased in C5-sufficient mice with histone injection.

### Extracellular histones promoted platelet aggregation and platelet/leukocyte aggregate (PLA) production

**Figure 6:**
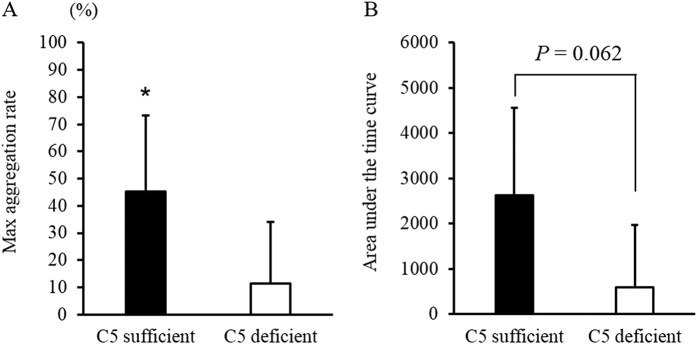
Extracellular histones induced platelet aggregation. Platelet-rich plasma (PRP) were collected from C5-suffcient and deficient mice. PRP were activated by the addition of histone for 10 min (final concentration of 900 μg/mL). The maximum aggregation rate and area under the time curve are shown in (**A**,**B**), respectively. The figures show the experimental means ± SD. *P < 0.05 vs group of C5- deficient (Student’s-t test).

**Figure 7:**
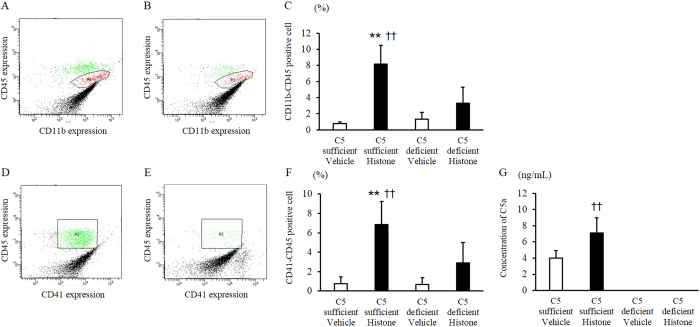
Extracellular histones increased the rates of CD41-CD45 complex (platelet/leukocyte aggregate) or CD11b-CD45 positive cells via the increasing of C5a. Mice were injected with histone or saline (45 μg/g, *n* = 6 per group). The blood samples were collected at 1 h after injection. The profiles of CD11b-CD45 positive cells and CD41-CD45 complex (platelet/leukocyte aggregates; PLAs) are shown in figure (**A** to **B**) or (**D** to **E**), respectively. The average rates are also shown in figure (**C** and **F**), respectively. The concentration of C5a are shown in figure (**G**). C5a was not detected in C5 deficient mice. The figures show the experimental means ± SD. **P < 0.01 vs Histone group of C5 deficient, ^††^*P* < 0.01 vs Vehicle group of C5 sufficient (Scheffe’s test).

The values for maximum aggregation rate and area under the time curve are shown in Fig. 6. Platelet aggregation in platelet-rich plasma (PRP) was promoted by incubation with extracellular histones. The maximum aggregation rate and area under the time curve were higher in C5-sufficient mice than in C5-deficient mice (maximum aggregation rate: *p* = 0.043, area under the time curve: *p* = 0.062) (Fig. 6A and B). The profiles of the CD41-CD45 complex (PLAs) and CD11b-CD45 positive cells are shown in Fig. 7. Extracellular histones increased CD11b expression and promoted PLA production. The rates of CD11b-CD45-positive cells (Fig. 7A to C) and PLAs (Fig. 7D to F) were significantly higher in C5-sufficient mice injected with histones than in C5-deficient mice. The complement component 5a (C5a) concentration in C5-sufficient mice injected with histones was significantly higher than that in C5-sufficient mice injected with saline (*p* = 0.024). In C5-deficient mice, C5a was not detected (Fig. 7G).

### C5a receptor antagonist decreased the toxicity of extracellular histones

**Figure 8:**
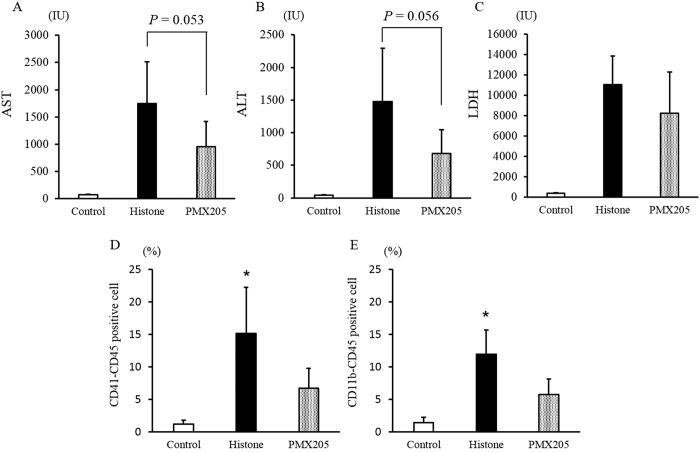
C5a receptor antagonist ameliorated the liver injury and decreased the rates of CD41-CD45 complex (platelet/leukocyte aggregate) or CD11b-CD45 positive cells. The mice were received a single tail-vein injection of C5a receptor antagonist PMX205 (50 μg/body) or saline. At 10 min after injection of saline or PMX205, the mice in the histone and PMX 205 groups were injected with unfractionated histones. The mice in the control group were injected with saline only. Liver function were defined as AST, ALT and LDH levels, and these are shown in (**A**–**C**), respectively. The profiles of CD41-CD45 complex (platelet/leukocyte aggregates; PLAs) and CD11b-CD45 positive cells are shown in figure (**D**,**E**), respectively. The figures show the experimental means ± SD. *P < 0.05 vs PMX205 group (Scheffe’s test).

The AST, ALT, and LDH levels are shown in Fig. 8A–C. The AST and ALT levels were lower in the PMX 205 group than in the histone group (AST: *p* = 0.053, ALT: *p* = 0.056) (Fig. 8A and B). Moreover, the C5a receptor antagonist decreased the PLA rates and CD11b expression (Fig. 8D and E).

## Discussion

Previous reports have indicated the pathogenic relationship between extracellular histones and thrombosis in animal models^8^,^11^,^14^. Extracellular histones promote cytotoxicity and thrombosis, which are associated with progression of DIC. To prevent or treat DIC, controlling both coagulation and inflammation systems is important. Although C5 is involved in the activation of these systems, its role in histone-induced lethal thrombosis has been unclear. Therefore, in this study, we investigated whether C5 promotes liver injury associated with histone-induced lethal thrombosis.

Activation of the coagulation system induces consumptive coagulopathy^2^. Coagulation disorders are triggers for multiple organ failure^2^,^3^. Coagulation disorders are characterized by prolongation of APTT and PT. In the present study, histone-induced lethal thrombosis and the coagulation disorder were milder in C5-deficient mice than in C5-sufficient mice. Since neutrophil extracellular traps (including histones) are digested by DNase-1^29^ and phagocytosis by macrophages^30^,^31^, extracellular histones are less likely to accumulate *in vivo*. However, excessive release of histones from cells can induce diverse physiological reactions. Extracellular histones induce thrombin generation through platelet aggregation^32^ and then activate the coagulation system. A previous study showed that DNA–histone (>357 AU) and dsDNA (>3.23 ng/mL) exhibited poor survival rates in the patients with DIC^9^. Activation of the coagulation system promotes cleavage of C5, and terminal complement components activate the coagulation system^22^,^23^. Our results support that crosstalk between C5 and coagulation systems promotes coagulation disorders.

Previous studies indicated that extracellular histones induce platelet aggregation^10^,^32^; some other reports suggested that C5a also promotes platelet aggregation^33^,^34^. In DIC patients, thrombocytopenia is induced by acute platelet aggregation, which promotes embolism. In our study, the aggregation rates in PRP were higher in C5-sufficient mice than in C5-deficient mice. In addition, histone-induced thrombocytopenia was milder in C5-deficient mice than in C5-sufficient mice. Our findings indicate that C5 is associated with histone-induced platelet aggregation. Notably, the PLA levels increased in the whole blood of C5-sufficient mice that had been injected with histones. Platelets and leukocytes form stable aggregates by binding to GPIIb and CR3 (CD11b)^35^. These aggregates promote the up-regulation and activation of CR3^36^. Platelets release growth factor for activating leukocytes^37^,^38^,^39^,^40^; thus, this complex induces endothelial damage in the acute phase of ischaemic events^41^. These reports indicate that PLAs comprise an important factor in the pathogenesis of vascular ischemic syndromes resulting in embolism. C5a is an anaphylatoxin generated from C5 cleavage via complement activation. A recent study has indicated that C5a production enhances PLA levels via increase in CD11b expression^42^. In the present study, the rates of CD11b-positive cells increased in C5-sufficient mice, and the C5a concentration was increased by extracellular histones. Furthermore, the C5a receptor antagonist prevented increase in CD11b expression and also decreased the PLA rate. These results indicated that C5a is associated with PLA production and are consistent with the results of a previous study^42^.

Xu *et al*. reported that histones induced fatal liver injury by releasing inflammatory cytokines^14^. However, the pathological mechanism underlying liver injury has been unclear. We found that C5 is associated with the embolism formation and liver function decline induced by histones. Furthermore, the hepatic blood flow decreased in C5-sufficient mice than in C5-deficient mice. These data indicate that C5 is associated with embolism-induced liver injury.

**Figure 9:**
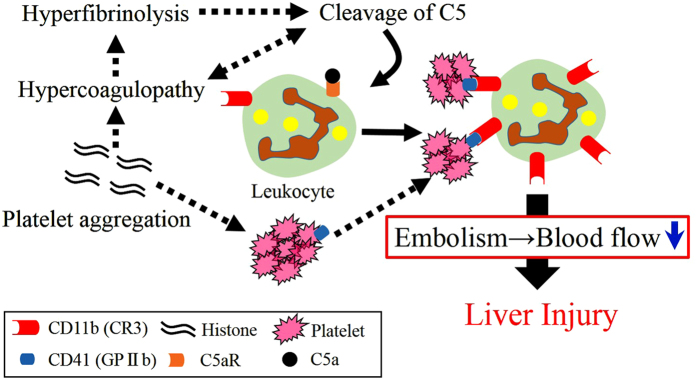
Summary for the mechanism of liver injury induced by histone. The black dotted arrow show previously known interactions of these systems. The black solid arrows identify the new paths in the present study. Histones induce platelet aggregation and coagulation disorder; the coagulation disorder promotes cleavage of C5. C5a increases the expression of CD11b (CR3). CR3 binds to CD41 (GPIIb), and this aggregations induce embolism. Embolism accelerates liver injury.

Previous studies have suggested that thrombin^17^ and factors IX, X, and XI promote C5 cleavage^21^. In addition, the coagulation cascade is promoted by activation of the complement system^19^,^20^,^22^,^23^. The present study suggested that C5a is a key molecule for lethal thrombosis induced by extracellular histones. In summary, histones induce platelet aggregation and coagulation disorder; the coagulation disorder in turn promotes C5 cleavage. C5a increases the expression of CD11b (CR3). To form stable aggregates, CR3 binds to CD41 (GPIIb), and this aggregation induces embolism (Fig. 9). Thus, C5 promotes liver injury associated with histone-induced lethal thrombosis. The present study indicates that blockage of C5 or C5a could be an appropriate therapeutic approach. Further studies are required to clarify the effects of these inhibitors.

## Methods

### Mice

All animal experiments were approved by the experimental animal board of Meijo University (Approval number: 2016-P-E-31) and conducted in compliance with the Animal Experiment Guidelines of Meijo University. The animals were maintained under conventional laboratory conditions and were given free access to food and water. Male DBA/1JLmsSlc and DBA/2CrSlc mice (Japan SLC, Shizuoka, Japan; age, 9–12 weeks) were used for the study of lethal thrombosis. DBA2/CrSlc mice are genetically deficient in complement C5^43^,^44^. DBA1/CrSlc mice have a normal complement system and were used as controls^43^,^44^. The lethal thrombosis model was prepared according to a previous study^8^. Briefly, mice received a single tail-vein injection of purified unfractionated histones from calf thymus (45–75 μg/g, Sigma-Aldrich, St Louis, MO), which contained little endotoxin. The total endotoxin doses received by the mice were 18.5 (45 μg/g histones), 24.6 (60 μg/g histones), and 30.8 ng/kg (75 μg/g histones). The mice were divided into three groups: high (75 μg/g)-, intermediate (60 μg/g)-, and low (45 μg/g)-dose groups. Saline was administered in the vehicle group. Six mice were included in each group and each time point in all experiments. After injection, the mice were monitored for survival up to 72 h. Since mice injected with high and intermediate doses had high mortality rates, we used a low dose of histone (45 μg/g) in the subsequent experiments. Under anaesthesia with isoflurane (Wako, Osaka, Japan), blood samples were collected at each time point to analyse the blood cell counts and liver function (aspartate aminotransferase [AST], alanine aminotransferase [ALT], and lactate dehydrogenase [LDH] levels).

### Blood coagulation test

Blood samples were collected from anaesthetized mice 1, 3, and 6 h after histone injection (45 μg/g). To prevent coagulation, blood was mixed with 3.13% (w/v) sodium citrate (Sigma). Plasma was prepared by centrifugation at 1500 × *g* for 10 min at room temperature (18–25 °C). PT and APTT were measured by standard methods, using a KC1 Delta automatic coagulation analyser with an electromechanical clot detection instrument (Trinity Biotech, Bray, Ireland).

### Blood counts and liver function

Blood samples were collected from anaesthetized mice 1, 3, 6, and 12 h after histone injection (45 μg/g). To prevent coagulation, blood was mixed with EDTA-2K (Dojindo Laboratories, Kumamoto, Japan). The WBC, RBC, and platelet counts were measured by Oriental Yeast Co. Ltd (Nagahama, Japan). To evaluate liver function, plasma was obtained by centrifugation of the remaining blood at 1500 × *g* at 4 °C for 10 min. AST, ALT, and LDH levels were also measured by Oriental Yeast Co. Ltd.

### Histological analysis of liver tissue

Liver samples were collected 6 h after histone injection (45 μg/g). For histological analysis, the liver was fixed in 10% buffered formalin (Japan Tanner Corporation, Osaka, Japan) and embedded in paraffin by conventional techniques. The sections (7-μm thick) were used for histological assessment by haematoxylin and eosin (H&E) staining. The images were captured at ×200 magnification under a BZ-X700 Fluorescence Microscope (Keyence, Osaka, Japan).

### Platelet aggregation test

The platelet aggregation test was performed as previously reported^45^. Briefly, blood samples were collected from anaesthetized mice without injection of histone or saline. Coagulation was prevented by addition of 3.13% (w/v) sodium citrate. PRP was prepared by centrifugation at 100 × *g* for 10 min at room temperature (18–25 °C). Platelet-poor plasma (PPP) was obtained by subsequent centrifugation of the remaining blood at 2000 × *g* for 10 min at room temperature (18–25 °C). Platelet aggregation was determined with an aggregometer (PAM-6C and PAM-8C; Mebanix Co. Ltd., Tokyo, Japan) on the basis of the method reported by Born and Cross^46^. PRP and PPP were pre-incubated at 37 °C for 1 min and then the platelets in the PRP were activated by the addition of histone (final concentration, 900 μg/mL). Platelet aggregation was evaluated on the basis of the maximum aggregation rate and area under the time curve for 10 min.

### Analysis of PLAs by fluorescent-activated cell sorting (FACS)

Blood samples were collected from anaesthetized mice 1 h after histone injection (45 μg/g). To prevent coagulation, blood was mixed with 3.13% (w/v) sodium citrate. Direct immunofluorescence staining was performed according to a previous report^41^ with a slight modification. In brief, blood (25 μL) was added to the microcentrifuge tube. To identify PLAs, the blood was mixed with monoclonal antibodies (Abs) against CD45, CD11b, and CD41. The antibodies were as follows: APC-conjugated rat IgG_2b_ anti-mouse CD45 (clone 30-F11; Biolegend, San Diego, CA), PE-conjugated rat IgG_2b_ anti-mouse CD11b (clone M1/70; Biolegend), and BV421-conjugated rat IgG_1_ anti-mouse CD41 (clone MWReg30; Biolegend) Abs. The isotype controls were APC-conjugated rat IgG_2b_ (Biolegend), PE-conjugated rat IgG_2b_, and BV421-conjugated rat IgG_1._ This mixture was gently stirred without vortexing and then incubated for 15 min at room temperature (18–25 °C) in the dark. Subsequently, the blood was fixed by adding lysing solution (Becton Dickinson, San Jose, CA) and incubation in the dark for 30 min. FACS analysis was performed using a BD LSR Fortessa X-20 system (Becton Dickinson) to detect expression changes on the cell surfaces.

### Enzyme-linked immunosorbent assay (ELISA) for measurement of C5a in plasma

Blood samples were collected from anaesthetized mice 1 h after histone injection (45 μg/g). To prevent coagulation, blood was mixed with EDTA-2K. The plasma was obtained by centrifugation of the remaining blood at 1500 × *g* at 4 °C for 10 min. To measure C5a levels in plasma 1 h after histone injection (45 μ*g*/g), a mouse C5a ELISA Kit (Abcam, Cambridge, UK) was used. All assays were analysed according to the manufacturers’ instructions. All samples were measured in duplicate and the mean value was used.

### Embolism analysis

ICG accumulates in the liver. To analyse liver blood embolism, the mice were administered ICG (10 μg/body, Wako) 1 h after histone injection. They were anaesthetized by isoflurane 10 min after intravascular injection of ICG. To analyse the liver tissue directly, the tissues were collected from mice. These images were acquired using the IVIS Spectrum system (Perkin Elmer Inc, Waltham, MA) and the fluorescent expression of ICG was quantified with the Living Image 3.2 software (Perkin Elmer Inc).

### Treatment with C5a receptor antagonist

To evaluate the effects of the C5a receptor antagonist in lethal thrombosis, the male DBA/1JLmsSlc mice were divided into three groups: control group, histone group in isotonic saline, and C5aR antagonist (PMX205) group. The mice received a single tail-vein injection of the C5a receptor antagonist PMX205 (Tocris Bioscience, Bristol) (50 μg/body) or saline. At 10 min after injection of saline or PMX205, the mice in the histone and PMX 205 groups were injected with unfractionated histones (45 μg/g). The mice in the control group were injected with saline only. To measure liver function and the PLA rate, blood samples were collected from anaesthetized mice 1 h after histone injection.

### Statistical analysis

Values have been provided in terms of mean ± standard deviation (SD). Comparisons among multiple groups were performed by analysis of variance (Kruskal-Wallis; Scheffe’s test). Analyses between two groups were performed by an unpaired t-test (Student’s t-test). In these tests, a two-sided value of *P* < 0.05 was considered significant. The SPSS v22.0 software (SPSS, Chicago, IL) was used for statistical analysis.

## Additional Information

**How to cite this article**: Mizuno, T. *et al*. Complement component 5 promotes lethal thrombosis. *Sci. Rep.* **7**, 42714; doi: 10.1038/srep42714 (2017).

**Publisher's note:** Springer Nature remains neutral with regard to jurisdictional claims in published maps and institutional affiliations.
